# The effect of solution-focused counseling on violence rate and quality of life of pregnant women at risk of domestic violence: a randomized controlled trial

**DOI:** 10.1186/s12884-021-03674-z

**Published:** 2021-03-20

**Authors:** Sepideh Dinmohammadi, Mohsen Dadashi, Elahe Ahmadnia, Leila Janani, Roghieh Kharaghani

**Affiliations:** 1 Department of Midwifery, School of Nursing and Midwifery, Zanjan University of Medical Sciences, Zanjan, Iran; 2 Department of Clinical Psychology, School of Medicine, Zanjan University of Medical Sciences, Zanjan, Iran; 3 Department of Biostatistics, School of Public Health, Iran University of Medical Sciences, Tehran, Iran

**Keywords:** Domestic violence, Solution-focused counseling, Quality of life, Pregnancy

## Abstract

**Background:**

Domestic violence during pregnancy as one of the most common social problems and major challenges of health systems can affect the health of the mother and fetus. The study aimed to compare the two groups of intervention based on solution-focused counseling and control in terms of violence and quality of life amounts in women who had experienced domestic violence.

**Methods:**

A randomized controlled trial on 90 pregnant women was blocked into two intervention groups (*n* = 45) and a control (*n* = 45). The intervention group received six counseling sessions with a solution-focused approach. Study tool included conflict tactics scale (CTS- 2) and short form health survey (SF-36). The tools were completed once before the intervention and again 6 weeks after the end of the counseling sessions by the participants of both groups. The *P*-value less than 0.05 was considered statistically significant.

**Results:**

A total of 82/90 pregnant women were analyzed. The results showed that median and interquartile range (IQR) of physical, psychological and sexual violence significantly decreased in the intervention group than the control group (Ps = 0.001). Moreover, quality of life scores significantly improved in the intervention group compared to the control group (*P* = 0.001).

**Conclusion:**

Solution-focused counseling could be an effective approach to reduce the amount of violence and increase the quality of life in women exposed to domestic violence.

**Trial registration:**

Iranian Registry of Clinical Trials IRCT2017040628352N4. Date of registration: August 20th 2017.

**Supplementary Information:**

The online version contains supplementary material available at 10.1186/s12884-021-03674-z.

## Background

According to the estimates published by World Health Organization (WHO), 35% of women worldwide are at risk of domestic violence and as many as 38% of women’s homicides are committed by their spouses [1]. The incidence of domestic violence against pregnant women is reported to be 52% in Iran [2] and 28% in Zanjan [3]. Domestic violence can take many forms among which the physical, psychological, and sexual violence are the main types [4]. Violence is commonly used in controlling and domineering women [5] which could happen by causing physical aggression or even death threats, physical assaults, activity limitation, denying women’s autonomy, and restricting women’s access to life facilities [6], this in return could cause physical and psychological traumas which would result in developing low self-esteem, hatred and fear from men [6, 7]. Spousal violence specifically during pregnancy as an additional threat to the mother and fetus health not only cause serious physical disorder but also causes serious fetal consequences such as abortion, pre-mature delivery, low birth weight, and low Apgar score at birth [8–10].

The quality of life scale especially in mental health area in women who had experienced violence is decreasing, while rates for mental health problems such as depression, anxiety, insomnia, violence, and suicide are rising among these women. The reasons for the declining quality of life among women who had experienced violence is psychological trauma caused by deprivation of liberty, verbal and emotional abuse caused by continuous insults, disregarding women’s emotions, social and economic inequalities, and doctorial actions of spouses, eventually would result in low confidence and self-esteem in women [11–13].

Empowering women plays a significant role in decreasing domestic violence which could consolidate the emotional bonds between couples [14, 15]; So, providing training and counseling sessions as an essential factor in this area is an important duty of health care workers [16]. Solution-focused brief counseling as one of the state-of-the- art and effective approaches in resolving marital problems was developed by a couple named Steve De Shazer and Insoo Kim Berg in Milwaukee brief family therapy center in Wisconsin, U.S.A., and is widely known as an ultra-modern brief therapy [1, 17]. This approach helps clients find solutions to their problems, in addition to that; it helps them in identifying their capabilities in a way to become more hopeful about the future and create useful changes in their positions and perspectives in life [18, 19]. The solution-focused approach claims that individuals are capable enough to promote the quality of their lives by creating appropriate solutions [20].

According to the searches conducted by researchers, there are no study on the effects of solution-focused approach on the quality of life in pregnant women who undergo violence in Iran or across the world. Thus, considering the high prevalence of violence, the harsh outcomes of violence during pregnancy and its impact on the quality of life of pregnant women, and due to the absence of similar studies conducted in Iran; the present study was conducted to determine the effects of solution-focused counseling on violence scale and the quality of life of pregnant women at risk of domestic violence.

## Methods

### Trial design

The study was a superiority parallel single-blind randomized controlled clinical trial with two groups of intervention and control in pregnant women at risk of domestic violence.

### Setting and participants

The inclusion criteria comprised minor and medium levels of domestic violence in physical, psychological, and sexual subscales of the CTS-2 in mothers, being over 18 years old, being less than or equivalent to 27 weeks of pregnancy based on Last Menstrual Period (LMP) or ultrasound results, literacy, living in the city of Zanjan, being married for at least a year, having no participation in any other classes or counseling courses simultaneously, having cell phones, absence of underlying illnesses, willingness to participate in counseling sessions and living together as a couple. The exclusion criteria included known psychological illness, psychoactive drug consumption, signs of addiction to drugs in pregnant women and their spouses, and being absent in more than one counseling sessions.

### Intervention

The recruitment and the intervention were done in childbirth preparation class in a comprehensive urban health care center in Zanjan, Iran. At the time of the study the center had the only governmental childbirth preparation classes in the entire city. Pregnant women learn about pregnancy, childbirth and breastfeeding, and do exercise related to pregnancy in these classes. In the intervention group, counseling sessions based on solution-focused approach were held weekly for a period of 6 weeks in the form of 90- min. Counseling sessions were conducted individually and were held by the researcher. The themes in each session were as follows: the aims of the first counseling session include highlighting general principles of solution-focused counseling and providing proper definitions of problems to clients. The second session focused on the familiarity of participants with the concept of quality of life and solution-focused approach. During the third session, clients learnt that there are different interpretations for an event and that they can develop the best interpretation in their minds while in the fourth session, clients were encouraged to discover exceptional opportunities of living as a couple. In the fifth session, with the help of miracle questions, participants were able to recognize their destructive behavior patterns. One of the main elements in this approach is the miracle questioning. This question actually encourages the person not to think about how and the chances of achieving the goals. Instead, the question arises as to what would occur to them if miraculous things happen, such as a miracle. This question helps people to have a very positive and different attitude towards their lives. With this question, one’s mind shifts its focus from the cause of what is happening. Instead, the mind goes in a direction where the person has nothing as a problem. In the sixth session, a conclusion was made from the whole previous sessions to help the clients replace and experience their former thoughts and behaviors with the new ones. The control group received no intervention. In order to prevent the exchange of information between the participants of the two groups, the intervention group was explained that the exchange of information related to the counseling sessions between the intervention and control groups could distort the results. Also the sessions for this group of participants were held in a different place. Six weeks after the last counseling session, both groups were invited to complete the study tool for the second time. At this time the session was conducted in the presence of an interviewer who was completely unaware of the grouping procedure. To comply with ethical issues, after the questionnaires were completed at the follow-up period, the sessions were held for individuals in the control group who were willing to participate in these sessions.

### Study outcomes

The outcomes of the present study included domestic violence and the quality of life in pregnant women who had been exposed to domestic violence which were measured and evaluated between the two intervention and control groups at 6 weeks follow ups.

### Sample size

Based on previous study [21], the sample size was calculated for each group of 45 people with an error of 5% and a power of 80%.

### Random allocation

Participants were selected through a convenience sampling method and divided into two intervention (A) and control (B) groups using, quadruple random blocks. At first, all sequences of participants in each block was considered and then 23 blocks sequence was selected from random table number. The overall number of 90 pregnant women entered into the study from which 45 individuals were allocated to intervention and 45 individuals to the control group.

### Data collection tools

Research tools for collecting data included demographic and reproductive checklist, CTS-2, and health-related quality of life questionnaire (SF-36); which were completed by qualified women. Demographic data included couple’s age, couple’s employment, couple’s residential address and education status, marriage longevity, economic status, having cell phones, number of marriages and having children from the previous marriage. Reproductive data related to pregnancy status included pregnancy duration, frequency of pregnancy and delivery, intended and unintended pregnancies, and infertility history. In order to identify whether pregnant women were at risk of domestic violence; CTS-2 was applied. The questionnaire is the newly revised version of the conflict tactics scale in addressing marital conflicts which was developed by Straus et el. (2007) with the confirmed Cranach’s Alpha of 79% for physical violence and 86% for psychological violence [22].

The applied questionnaire in the present study was validated by Ardabily et el. (2011) with the Cronbach’s Alpha of 80% [23] which comprised 36 questions studying the conflict tactics on different dimensions of negotiation, physical, psychological, and sexual violence that leads to injuries. Although, the applied criteria in measuring the frequency and severity of violence in the CTS-2 is considered to be from last year, according to the questionnaire designer, 1 year is predetermined and could be generalized to the desired time expected by the researchers [22]. Thus, in the questionnaire associated with the present study, instead measuring the previous year, 3 months’ period was accounted for measurement. The SF-36 was applied for measuring the quality of life of participants, which included 36 questions and its validity in Iran has been verified by Montazeri et el. (2005) with the Cronbach’s Alpha of 77 to 90%. This tool evaluates the quality of life in two dimensions of physical and psychological health. Each dimension contains four subscales. Physical subscales include physical function, role limitation-physical health, bodily pain and general health and psychological dimension include vitality, psychological health, role limitation-emotional health, and social functioning [24].

### Statistical analysis

The collected data were analyzed using SPSS V.16 and R statistical software. In order to study the normal distribution of variables, Kolmogorov– Smirnov test was applied. Categorical variables were examined using the Chi-Squared test and Fisher’s exact test. In order to compare conflict tactics scale and the quality of life subscales between the two groups of intervention and control at baseline, independent t-test and Mann- Whitney U test were used. Since the residual distribution of variance between the two groups was not normal and there were no parametric covariance analysis (ANCOVA) assumptions, nonparametric ANCOVA was used to compare the variables between the two groups after the intervention. In this test, the outcomes are compared between the groups adjusted for the amount of baseline variables. Also, for comparing the conflict tactics scale, the quality of life, and their subscales between different phases of study, Wilcoxon and paired t-test were applied. The significance level of the aforementioned tests was based on *P* value < 0.05. The economic class was calculated considering factors such as house foundation, housing, and transportation means. In addition to that, for calculating social and economic levels, factors such as job ranking, education, and economic class were taken into consideration.

## Results

### Participants

**Fig. 1:**
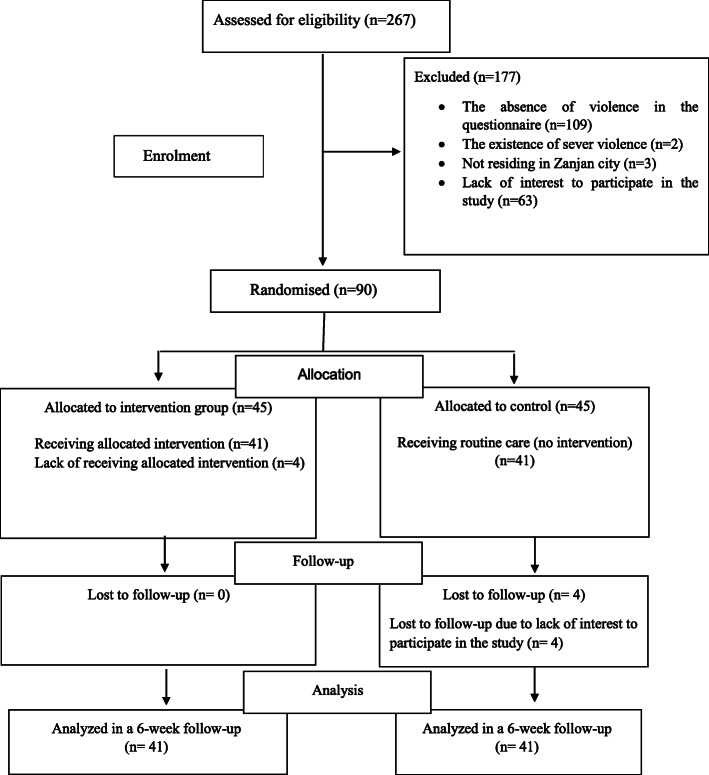
The flowchart of sampling

Overall, 267 pregnant women completed the conflict tactic questionnaire. Of them 158 individuals had minor and medium levels of domestic violence based on conflict tactics scale (CTS-2) (the violence rate 59%). Sixty-eight women did not meet the inclusion criteria. Therefore, 90 pregnant women entered into the study and randomly assigned to the intervention and control groups. In the intervention group four participants were excluded from the study, due to not attending the sessions (*n* = 4). Also, in the control group four participants were excluded due to unwillingness to continue the study. Eventually, the data from 82 pregnant women was analyzed. Figure 1 shows the flow diagram of the participants during the study. Others who had domestic violence but were not eligible or excluded from the study were referred to psychologist’s offices.

### The preliminary analysis

The mean (standard deviation) for women’s age was 27.55 (5.13) in the intervention group and 27.26 (4.46) in control group, and as for men; the mean (SD) of the men’s age was 31.44 (5.72) in the intervention group and 31.82 (4.72) in the control group. The mean (SD) for gestational age was 22.6 (2.35) in the intervention group and 22.73 (2.41) in control group. The majority of the participants socially and economically belonged to third class. The median of the gravidity in both groups was one. The two groups were similar with respect to age, job, education, gestational age, parity, duration of marriage, wanting pregnancy, economic and social classes (Table 1).

**Table 1:** Comparison of demographic and obstetric data in intervention and control groups

Variable	Intervention (*n* = 45)	Control (*n* = 45)
	Median (IQR)	Median (IQR)
Woman’s age (year)^a^	27.55 (5.13)	27.26 (4.46)
Man’s age (year)^a^	31.44 (5.72)	31.82 (4.72)
Female education (year)	14 (5.5)	12 (4)
Male education (year)	12 (5)	12 (5)
Duration of marriage (year)	3 (8)	3 (5)
Woman’s job (housekeeper)^b^	42 (93.3)	42 (93.3)
Previous marriage (yes)^b^	1 (2.2)	1 (2.2)
Child from previous marriage (no)^b^	45 (100)	45 (100)
Economic class^b^		
First	8 (17.8)	12 (26.7)
Second	17 (37.8)	10 (22.2)
Third	12 (31.1)	14 (26.7)
Fourth	7 (15.6)	8 (17.8)
Fifth	1 (2.2)	1 (2.2)
Social class^b^		
First	5 (11.1)	6 (13.3)
Second	14 (31.1)	12 (26.7)
Third	19 (42.2)	18 (40)
Fourth	6 (13.3)	9 (20)
Fifth	1 (2.2)	0
Gestational age at enrollment (week)^a^	22.6 (2.35)	22.73 (2.41)
Wanting pregnancy^b^		
Wanted	12 (26.7)	15 (33.3)
Unplanned	30 (66.6)	30 (66.7)
Unwanted	3 (6.7)	0
Infertility history (yes)^b^	1 (2.2)	4 (8.9)
Gravidity	1 (1)	1 (1)
Parity	0 (1)	0 (1)
Duration of infertility (year)	1	0

^a^Mean (standard deviation), ^b^Numbers (%)

### The intervention effects on violence

The most observed violence in this study was associated with psychological violence. The median and interquartile range (IQR) of psychological violence was 0.375 (0.62) in the intervention and 0.25 (0.38) in the control group. Also, the median (IQR) of minor physical violence in both groups was 0 (0.08). The groups were similar with respect to the conflict tactics subscales (Table 2).

**Table 2:** Comparison of the subscales of conflict tactics scales between intervention and control groups at baseline

Variable	Intervention (*n* = 45)	Control (*n* = 45)	*p*-value
	Median (IQR)	Median (IQR)	
Negotiation	2.1 (1.7)	1.83 (1.8)	0.715 ͣ
Psychological abuse	0.375 (0.62)	0.25 (0.38)	0.068^b^
Minor physical violence	0 (0.08)	0 (0.08)	0.431^b^
Severe physical violence	0 (0)	0 (0)	0.980^b^
Sexual abuse	0 (0.50)	0 (0)	0.096^b^

ͣ Independent t test

^b^ Mann-Whitney U test

The median (IQR) score of psychological violence in the intervention group had decreased dramatically from 0.375 (0.62) at baseline to 0 (0.25) at follow up. Also, the median (IQR) score of the minor physical violence subscale at 6 weeks follow-up in the intervention group was 0 (0) which in comparison to the median baseline value of 0 (0.08), there was a statistically significant decreased. After matching the nonparametric ANCOVA test with the conflict tactics scale variables at the baseline; the results indicated that there was a significant difference between the two groups in negotiation (*P* < 0.001), physical violence (*P* = 0.001), psychological violence (*P* = 0.001), and sexual violence (*P* = 0.001), however with regards to severe physical violence, no significant difference had been observed at the follow- up period (Table 2).

### The intervention effects on quality of life

The baseline median (IQR) of the quality of life in the intervention group was 65 (27.8) and in the control group was 75.6 (22.5). The intervention group had reasonable lower quality of life scores than the control group at baseline. However, it wasn’t statistically significant it may show that they were in worse situation than the control group. In the present study, the highest subscales scores were associated with physical functioning (The median (IQR) of the physical functioning in the intervention group was 80 (30) and in the control group was 85 (20)). The groups were similar with respect to the quality of life and its components (Table 3).

**Table 3:** Comparison of quality of life and its sub-scales between two intervention and control groups during different phases of the study

	Variable	Intervention	Control	***P***-value
		Median (IQR)	Median (IQR)	
**Baseline**	Quality of life	65 (27.8)	75.6 (22.5)	0.052 ͣ
	Vitality	55 (25)	65 (27.5)	0.092 ͣ
	Mental health	68 (26)	72 (28)	0.184 ͣ
	General health	65 (25)	70 (27.5)	0.153 ͣ
	Bodily pain	67.5 (20.8)	67.5 (32.5)	0.227^b^
	Physical functioning	80 (30)	80 (20)	0.188^b^
	Role limitation-physical health	75 (62.5)	100 (25)	0.146^b^
	Role limitation-emotional health	66.55 (100)	66.66 (66.66)	0.126^b^
	Social functioning	75 (25)	75 (37.5)	0.358^b^
**Follow-up**	Quality of life	73.2 (21.2)	74 (20.3)	0.001^c^
	Vitality	70 (25)	65 (27.5)	0.003^c^
	Mental health	76 (24)	72 (28)	0.004^c^
	General health	70 (20)	75 (20)	0.247^c^
	Bodily pain	70.66 (16.2)	70.7 (21.27)	0.014^c^
	Physical functioning	85 (25)	80 (20)	0.023^c^
	Role limitation-physical health	75 (50)	75 (50)	0.134^c^
	Role limitation-emotional health	66 (67)	100 (77)	0.541^c^
	Social functioning	75 (12.5)	75 (25)	0.019^c^

^a^Independent t test

^b^ Mann-Whitney U test

^c^ Nonparametric ANCOVA

The median (IQR) of the quality of life at follow-up was 73.2 (21.2) in the intervention group which indicated a statistically significant increase in comparison to the baseline value of 65 (27.8). However, there was a significant difference between the two groups with regards to total quality of life (*p* = 0.001), vitality (*p* = 0.003), psychological health (*p* = 0.004), bodily pain (*p* = 0.014), physical functioning (*p* = 0.023), and social functioning (*p* = 0.019) at the follow- up period, however, there was no significant difference in other subscales of general health, role limitation-physical and role limitation-emotional health (Table 3).

In the intervention group, the changes of the conflict tactics subscales of negotiation (*p* < 0.001), physical violence (*p* = 0.039), psychological violence (*p* < 0.001), and sexual violence (*p* < 0.001) at follow-up phase versus baseline was statically significant. However, no statistically significant difference was recorded for severe physical violence.

Also, there were significant changes in quality of life subscales including general quality of life (*p* < 0.001), vitality (*p* = 0.002), psychological health (*p* < 0.001), general health (*p* = 0.003), role limitation-emotional health (*p* = 0.006), and social functioning (*p* = 0.045) at the follow-up period in comparison to baseline phase. However, there were no significant statistical differences in areas of bodily pain, physical functioning, and role limitation-physical health.

## Discussion

The results of the study showed that, the rate of psychological, physical and sexual violence in the intervention group decreased compared to the control group. The basics of the solution-oriented approach are empowerment and increasing self-confidence. It seems that women, who underwent this type of counseling, found that they are able to find appropriate solutions to their problems and gained the ability to solve problems through positive negotiations with their spouses. Therefore, the amount of physical, psychological and sexual violence decreased. In the rate of severe physical violence, no statistically significant change was observed between the intervention and control groups in the follow-up. This was due to the fact that no new cases of this type of violence were observed in any of the participants of the intervention and control group. Since the follow-up period in this study was in the third trimester of pregnancy, it was possible that with increasing gestational age and increased risk of complications in the third trimester, spouses were less likely to commit this type of violence. The most common type of violence was psychological violence. This result was consistent with studies conducted in descriptive researches in Iran and the world [25–27]. The effect of counseling in reducing violence scores had indicated that raising awareness and empowering women would reduce the rate of violence [28]. In line with this research, other studies have also shown that cognitive counseling and anger management training are effective approaches to reduce domestic violence [29, 30].

The intervention group had reasonable lower quality of life scores than the control group at baseline. However, it wasn’t statistically significant it may show that they were in worse situation than the control group. Align with the findings of the current research, other studies has shown that solution- focused counseling can increase the quality of life in women [20]. Furthermore, solution-focused counseling improves the quality of marital relationship between the couples and is effective on depression and satisfying about marital life [31, 32]. Also, conflict tactics counseling can lead to the reduction of marital conflicts between the couples [33]. The decline of violence rate and the increase of positive negotiations are two of the most likely reasons accounted for the increase of the quality of life in women who had undergone counseling and this has brought more emotional support from spouses.

The results of the current study indicated that the solution-focused approach can be an appropriate method to treat domestic violence and improve the quality of life dimensions in women who have experienced violence and are eligible for counseling.

### Strengths and limitations

One of the main strengths of this study was the conduct of counseling intervention based on a new approach to controlling domestic violence against women. In which all the principles related to clinical trial studies such as one-sided blindness, random allocation and concealment of allocation were observed. The present study had some limitations. For example, because the questionnaires were completed on a self-report basis, it was likely that women did not respond correctly to the questionnaire for fear of revealing their private life issues. Therefore, to alleviate this concern, participants were given the necessary assurance that the information was confidential. Another limitation of the present study was the short follow-up period due to the limited time of pregnancy.

## Conclusion

Solution-focused individual counseling has been found to be significantly effective in reducing domestic violence and increasing the quality of life in the intervention group and has led to improvements following negotiation and reduction in physical, psychological, and sexual violence. Also, the quality of life scores in other dimensions such as vitality, psychological health, bodily pain, physical and social functioning had been improved; however, still some domain of functioning such as the reduction of severe physical violence, improvement of general health components, role limitation-physical health and role limitation-emotional health remained unchanged. Due to the reduction of the majority of violence components and general quality of life; the implementation of this approach by healthcare institutions or other organizations in contact with women at risk of violence is approved to be beneficial. According to the results of the current study, there hasn’t been a significant increase on the quality of life subscales such as general health, role limitation-physical health and role limitation-emotional health. It is better to use a larger and more diverse population for future studies whereby through extended follow-up periods; a thorough evaluation of counseling results would be possible. Also, it would be wise to have similar studies conducted on non-pregnant women.

## Supplementary Information

**Additional file 1.** Questionnaire.

## Data Availability

The dataset used in the present study is available from the corresponding author upon reasonable request.

## Abbreviations

CTS-2: Conflict Tactics Scales
SF-36: Short Form health survey
LMP: Last Menstrual Period
