# Supplementary material for: The effect of solution-focused counseling on violence rate and quality of life of pregnant women at risk of domestic violence: a randomized controlled trial
Source: BMC Pregnancy Childbirth. 2021 Mar 20;21:221. doi: 10.1186/s12884-021-03674-z (PMC7981895; doi:10.1186/s12884-021-03674-z)
Supplement: Supplementary file 1 — Additional file 1. Questionnaire. [file 12884_2021_3674_MOESM1_ESM.docx]

**Supplementary file**

**questionnaire**

CTS-2 is the revised version of the conflict tactics scale in addressing marital conflicts which was developed by Straus et el. (1996) with the confirmed Cranach's Alpha of 79% for physical violence and 86% for psychological violence.

Murray A. Straus. Conflict Tactics Scales. Encyclopedia of Domestic Violence. 2007; 44: 190- 7.

The applied questionnaire in the present study was validated by Ardabily et el. (2010) with the Cronbach's Alpha of 80%.

Ardabily HE, Moghadam ZB, Salsali M, Ramezanzadeh F, Nedjat S. Prevalence and risk factors for domestic violence against infertile women in an Iranian setting. Int J Gynaecol Obstet. 2011; 112 (1):15-7. https://doi. 10.1016/j.ijgo.2010.07.030
Short form 36 health survey questionnaire (SF-36) was applied for measuring the quality of life of participants, which included 36 questions and its validity in Iran has been verified by Montazeri et el. (2010) with the Cronbach's Alpha of 77% to 90%. This tool evaluates the quality of life in two dimensions of physical and psychological health.

Montazeri A, Goshtasebi A, Vahdaninia M, Gandek B. The short form health survey (SF-36): translation and validation study of the Iranian version. Qual Life Res. 2005; 5 (1): 49- 56. https://doi.10.1007/s11136-004-1014-5.
